# Thin Film Composite Polyamide Reverse Osmosis Membrane Technology towards a Circular Economy

**DOI:** 10.3390/membranes12090864

**Published:** 2022-09-07

**Authors:** Amaia Lejarazu-Larrañaga, Junkal Landaburu-Aguirre, Jorge Senán-Salinas, Juan Manuel Ortiz, Serena Molina

**Affiliations:** 1 IMDEA Water Institute, Avenida Punto Com, 2, Alcalá de Henares, 28805 Madrid, Spain; 2 BETA Tech. Center, University of Vic-Central University of Catalonia, Ctra. de Roda, 70, 08500 Vic, Spain

**Keywords:** reverse osmosis, end-of-life, circular economy, eco-design, fouling, membrane recycling and reuse, indirect recycling, energy recovery, life cycle assessment

## Abstract

It is estimated that Reverse Osmosis (RO) desalination will produce, by 2025, more than 2,000,000 end-of-life membranes annually worldwide. This review examines the implementation of circular economy principles in RO technology through a comprehensive analysis of the RO membrane life cycle (manufacturing, usage, and end-of-life management). Future RO design should incorporate a biobased composition (biopolymers, recycled materials, and green solvents), improve the durability of the membranes (fouling and chlorine resistance), and facilitate the recyclability of the modules. Moreover, proper membrane maintenance at the usage phase, attained through the implementation of feed pre-treatment, early fouling detection, and membrane cleaning methods can help extend the service time of RO elements. Currently, end-of-life membranes are dumped in landfills, which is contrary to the waste hierarchy. This review analyses up to now developed alternative valorisation routes of end-of-life RO membranes, including reuse, direct and indirect recycling, and energy recovery, placing a special focus on emerging indirect recycling strategies. Lastly, Life Cycle Assessment is presented as a holistic methodology to evaluate the environmental and economic burdens of membrane recycling strategies. According to the European Commission’s objectives set through the Green Deal, future perspectives indicate that end-of-life membrane valorisation strategies will keep gaining increasing interest in the upcoming years.

## 1. Introduction

Climate change, population and economic growth, and the increasing anthropogenic pollution of water resources have exacerbated water scarcity. As a consequence, water availability (in terms of quantity and quality) has become one of the most challenging issues facing humanity in the 21st century [1,2]. 

Under this scenario, sea and brackish water desalination have evolved as essential processes to support the economic activity in water-scarce areas, producing high-quality water for irrigation, domestic, or industrial usage. In 2019, the total number of installed desalination plants was reported as 21,123, with a freshwater production capacity of approximately 126.57 million m^3^·day^−1^ [3]. Among other desalination technologies, RO is currently the most energy-efficient process developed at an industrial scale [4]; consequently, it dominates the desalination market, accounting, in 2018, for 84% share of the total number of operational desalination plants and entailing 69% of the total desalinated water produced in the world [5]. Furthermore, RO desalination is forecasted to grow at a constant Compound Annual Growth Rate (CAGR) of around 10.3% over the period 2020–2025 [6]. 

Due to fouling and chemical degradation of the membrane performance, it is estimated an annual membrane replacement rate ranged from 5%, in the case of Brackish Water RO desalination (BWRO) to 20% in Sea Water RO desalination (SWRO) [7]. Combined with the installed desalination capacity, as a trade-off, it was estimated that by 2025, more than 2,000,000 End-of-Life (EoL) RO modules will be yearly discarded in the world (>32,000 tonnes of plastic waste generation) [8]. Nowadays, these modules are commonly disposed of in landfills or, less frequently, incinerated for the recovery of energy [9]. Hereby, owing to the current non-biodegradable petroleum-based RO module composition, plastic waste generation and management represent critical challenges for the sustainability of the RO desalination industry.

To increase the sustainability of membrane-based technologies, it is essential to integrate the complete life cycle of RO modules into a circular economy model. Circular economy is an economic system that replaces the EoL concept with reducing, alternatively reusing, recycling, and recovering materials, closing the loops of materials and energy, with the aim to reach sustainable development in terms of environmental quality, economic prosperity, and social equity, enabled by the creation of new business models [10]. In this context, the directive 2008/98/EC on waste and its amendment of 2018 (directive 2018/851) [11,12] established for the first time a waste management hierarchy based on the 4R’s principle (Reduce, Reuse, Recycle, and Recovery) and identified landfill disposal as the least sustainable waste management practice. Afterwards, in 2019, the European Green Deal detailed a number of transformative policies to foster the transition to sustainable economic growth, with the ambitious commitment to be the first climate-neutral continent by 2050 [13]. The mobilisation of the industry for a clean and sustainable activity is one of the main strategic areas that were identified in the Green Deal. An important pathway in this regard is the development of longer-lasting products that can be repaired, recycled, and reused. In concordance, research and innovation are fundamental keys to advancing toward a cleaner industry. Furthermore, in 2020, the Circular Economy Action Plan [14] was adopted as one of the main building blocks of the Green Deal, detailing sustainable initiatives along the entire life cycle of products (i.e., design, maintenance, reuse, and recycling). 

**Figure 1:**
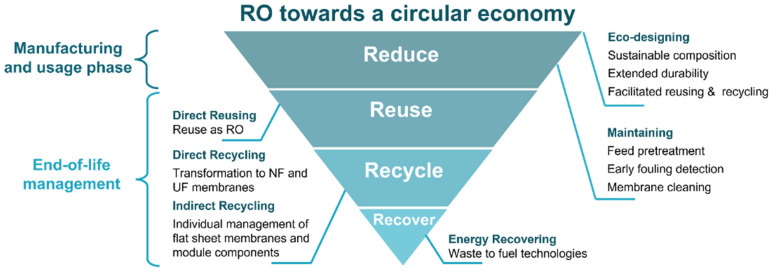
Possible pathways of waste hierarchy to be implemented by membrane technology.

In agreement with the abovementioned, the present review aims to contextualise the latest advances and future challenges for the integration of RO membrane technology in a circular economy. For that purpose, the entire life cycle of the membrane has been analysed, from membrane design and manufacturing to EoL membrane management (Figure 1). 

Hereof, Section 2 is dedicated to envisaging a greener RO membrane composition based on biopolymers, recycled materials, and green solvents, which would help to reduce the use of hazardous chemicals while improving the biodegradability and recyclability of the membranes. In addition, innovative membrane synthesis and modification methods for improving fouling and chlorine resistance are reviewed. Likewise, the importance of replacing the actual petroleum-based polymeric module components is discussed. Thereafter, feed pre-treatment technologies, early fouling detection methods, and membrane cleaning protocols for the prevention and mitigation of fouling during the usage phase are reviewed. Section 3 analyses current membrane management patterns and reviews recent advances in EoL membrane reuse, recycling, and energy recovering alternatives, giving special attention to emerging indirect recycling strategies which enable the sorting and recycling of individual membranes and module plastic components. Section 4 critically analyses the potential application of Life Cycle Assessment (LCA) as a tool to evaluate the environmental impact associated with EoL membrane management strategies previously analysed, helping in the decision-making process. Lastly, Section 5 discusses the major challenges and future prospects of the integration of RO membrane technology in a circular economy. 

## 2. Manufacturing and Usage Phase

### 2.1. Current RO Membranes and Modules

Currently, around 90% of commercially available RO membranes are Thin Film Composite Polyamide (TFC-PA) membranes [15,16]. TFC membranes are those assembled with several layers of diverse polymeric materials. In the case of TFC-PA RO membranes, an ultrathin, dense polyamide layer (PA, ~0.2 µm thick, pore size < 1 nm) acts as the selective layer, supported by a thicker porous layer, such as polysulfone (PSF, 40–150 µm thick), and a considerably thicker layer as mechanical support, such as non-woven polyester (PET, 120–150 µm thick) [17]. The PSF layer is commonly prepared on top of the PET non-woven layer by the phase inversion method using organic solvents (e.g., dimethylformamide [DMF]; dimethylacetamide [DMA], N-methyl-2-pyrrolidone [NMP]) as the solvent phase and water as the non-solvent phase. Then, the thin selective PA layer is assembled mostly by the interfacial polymerisation of organic diamines (e.g., m-phenylenediamine) and carboxylic acid monomers (e.g., trimesoyl chloride), typically using water as a non-organic solvent and hexane as an immiscible organic solvent [18]. It is estimated that current membrane fabrication processes generate approximately between 100 and 500 L of wastewater per square metre of membranes produced [19]. Such wastewater contains certain amounts of the toxic organic employed in the manufacturing process (e.g., DMF, DMA, NMP), which requires advanced treatments before disposal [19].

**Figure 2:**
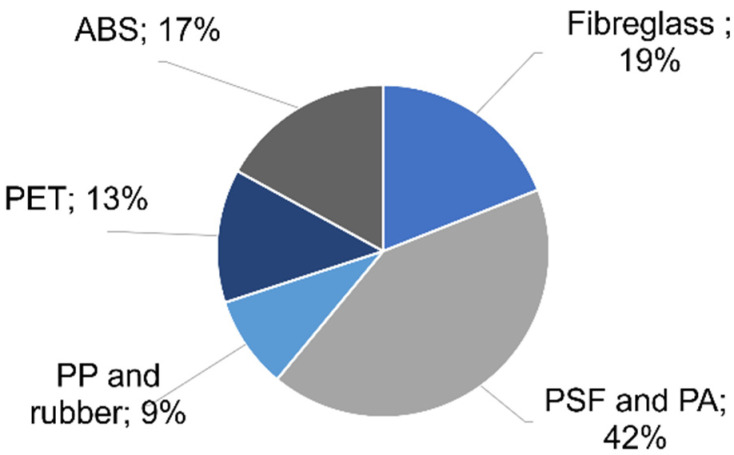
Composition (in percentage by weight) of a conventional 8″ RO module. ABS, acrylonitrile butadiene styrene; PET, polyester; PP, polypropylene; PSF, polysulfone; PA, polyamide, rubber, and fibreglass.

At an industrial scale, RO membranes are commonly fabricated in a spiral wound module configuration with the objective to increase the membrane area in a reduced space and to confer to the module of high-pressure resistance [20]. Therefore, apart from the RO membranes, the RO module incorporates other kinds of polymeric materials, such as acrylonitrile butadiene styrene (ABS), polypropylene (PP), polyester (PET), rubber, fibreglass, and some glued parts containing epoxy-like components. In this work, the membrane composition by weight was measured and the results are shown in Figure 2. These results are in good agreement with that previously published in [21].

As it can be observed, RO modules have a non-biodegradable composition based on petroleum-based polymers and composites. As long as the lifespan of RO membranes is often limited between 5 to 10 years, it is crucial to improve the biodegradability, reusability and recyclability of the membranes and module components in order to reduce landfill disposal.

### 2.2. Implementation of Eco-Design Principles in RO Module Manufacturing

**Figure 3:**
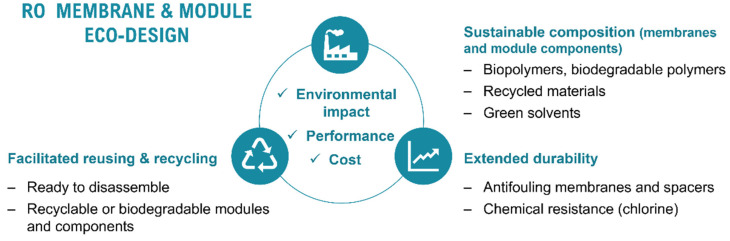
Main aspects to be considered in RO membrane and module eco-design.

It is estimated that 80% of the environmental impacts associated with a product could be reduced at the design stage [22]. Eco-design involves the consideration of all the environmental impacts related to a product’s lifespan (i.e., manufacturing, usage, and EoL management), along with other conventional considerations (i.e., performance and cost) (Figure 3). 

#### 2.2.1. Sustainable Composition

Biopolymers are defined as those polymers based either on renewable sources, such as plants (e.g., cellulose-based [23], isosorbide [24]), animals (e.g., polylactic acid [25], polyhydroxyalkanoates [26]), microorganisms (e.g., chitosan [27]), or those based on petroleum sources but that are biodegradable (e.g., polycaprolactone [28]) and thus undergo a complete degradation after their intended purpose by bacterial decomposition processes [29]. Research on the implementation of biobased polymers for membrane preparation has been mainly limited to microfiltration (MF) [26], ultrafiltration (UF) [23,24,27,28], and pervaporation [25] membranes, and fewer examples can be found in the preparation of biobased RO membranes. Interestingly, the first RO membranes (developed in the 1960s) were composed of cellulose acetate, a biopolymer (and thus biodegradable) synthetised from cellulose [23]. However, cellulose acetate membranes are damaged by high and low pH values, therefore limiting RO applications. To overcome this issue, the use of chitosan as a renewable biopolymer for the preparation of RO membranes was proposed in the 1980s [30]. Chitosan is a biopolymer closely related to cellulose that can be extracted from shellfish wastes in the form of chitin and converted to chitosan by deacetylation [31]. The abovementioned RO membrane attained a rejection coefficient of monovalent salts of 79% [30]. Biosourced polyesters (PES) have recently demonstrated high performance for nanofiltration membrane fabrication [32]. Biosourced PESs can be dissolved using green solvents (e.g., PolarClean). Furthermore, aside from being biodegradable, the resulting membrane is fully recyclable in such a way that monomers can be recovered by chemical recycling. Aside from biopolymers, bamboo fibres have been demonstrated to improve the mechanical stability of the membrane support in a completely biobased polylactic acid/bamboo fibre membrane [33].

Polymer blending is a strategy to improve the properties of a material, aiming at its optimisation for a certain application [34]. Adjusting the polymer blend ratio could allow for fine-tuning the molecular sieving properties of the membranes [35], reducing the free volume and freeing hydrophilic groups of biopolymers (e.g., chitosan) [36]. Moreover, crosslinking polymer blends increases the hydrophilicity of the resulting membranes [37]. In such a way, RO membranes fabricated with poly(vinyl alcohol)/chitosan blends crosslinked with silane demonstrated higher water flux than non-crosslinked membranes (0.78 L m^−2^ h^−1^ and 1.84 L m^−2^ h^−1^, respectively), with a monovalent salt rejection of 80% [37]. 

Emerging studies suggest the use of recycled/upcycled materials for membrane fabrication and modification [38]. For instance, biopolymers such as cellulose, hemicellulose, and lignin can be recovered from the waste biomass of the agriculture, food, and beverage industries, etc. [39]. In this line, A Alammar et al. [40] dissolved date seed biomass using green solvents (i.e., ionic liquids and dimethyl sulfoxide) to prepare biodegradable membranes. However, the membranes require additional coating layers of polydopamine (PDA) (added by layer-by-layer assembly) to attain the rejection of the target solutes (methyl orange and acid fuchsin). In such a way, after the addition of four PDA coating layers, with a deposition time of 24 h each, a rejection of 83% was obtained. H Li et al. [41] coated a polyvinylidene fluoride (PVDF) membrane by filtering (pump vacuum filtration) a water-based polyurethane solution containing pine nut shell powder, obtaining a superoleophobic membrane intended for oil-in-water separations. The membrane achieved a separation efficiency of oil-in-water emulsions (e.g., crude/water and petroleum ether/water mixtures) of >99% and showed a good adsorption capacity of water-soluble dyes and heavy metal ions, thus demonstrating potential applicability for industrial oil-bearing wastewater treatment. In a different way, J. Cavalcante et al. [42] proposed a method to enable the chemical recycling of disposable face masks, widely used as a consequence of the COVID-19 pandemic (~10 million masks per month, equivalent to 30–40 tons of plastic waste [43]). The method for upcycling this waste consisted of dissolving the face masks using green solvents and obtaining recycled polypropylene for the preparation of a free-standing solvent-resistant membrane, with a rejection capacity of 98% for roxithromycin and rose bengal. 

Among other types of wastes, the potential of EoL membranes to be recycled as a mechanical support for membrane fabrication should be highlighted. EoL membranes are thin films with outstanding mechanical and chemical resistance and an implicit low cost. Apart from EoL RO membrane recycling, which is deeply analysed in Section 3.2 and Section 3.3, the upcycling of low-pressure EoL membranes into high-pressure membranes could be attained by several membrane fabrications and modification methods. In this line, R. Dai et al. [44] employed fouled EoL MF membranes for the fabrication of TFC-PA NF membranes by interfacial polymerisation. The biopolymers (foulants) in the EoL membrane acted as additional channels for water transport, reaching a high flux (~30 L m^−^^2^ h^−^^1^ bar^−^^1^) and a high Na_2_SO_4_ rejection (95%).

Regarding the use of solvents, ideally, manufacturing methods which completely avoid the use of solvents or the use of water as a universal solvent should be preferentially employed [45]. However, up to now, membrane manufacturing methods have been commonly based on polymeric solutions, requiring considerable amounts of organic solvents, and solvent-free membrane preparation studies have been practically limited to the case of MF membranes (e.g., ceramic MF membranes prepared by extrusion [46], ceramic photocatalytic MF membranes prepared by a solvent-free sol-gel method [47]). In the case of UF and NF membranes, the use of water-soluble polyelectrolytes has been reported as a greener alternative to commonly used organic solvents [48]. However, water cannot dissolve uncharged polymers commonly used in membrane manufacturing. In those cases greener alternatives to currently used organic solvents include biosourced solvents (e.g., γ-valerolactone, glycerol [49]), non-synthetic organic solvents (e.g., Rhodiasolv^®^ PolarClean [32,49]) and non-volatile ionic liquids [45,50]. For instance, it has been demonstrated that cellulose acetate (a widely used biopolymer for the fabrication of RO membranes in the 1960s) could be dissolved using several green solvents, including dimethyl sulfoxide/acetone, PolarClean, methyl lactate, triethyl phosphate, and ionic liquids [45,50,51]. In this manner, the sustainability of RO membranes could be essentially improved. In the case of current TFC-PA RO membranes, the replacement of currently used organic solvents with ionic liquids allowed for a 20-fold reduction in m-phenylenediamine concentration and avoided the use of surfactants and catalysts in [52].

As was described above, a novel biobased composition could result in performance trade-offs (e.g., the lower salt rejection capacity exhibited by chitosan and poly(vinyl alcohol)/chitosan membranes in respect to current PA-TFC membranes [33,37]). In addition, biobased membranes commonly show poor mechanical properties, which could represent a limitation for practical applications [23]. Overall, a great academic and industrial effort will be required to reach the performance and properties of current commercial TFC-PA RO membranes.

#### 2.2.2. Extended Durability

Extending the service time of the membranes is the first step in the waste management hierarchy [53]. The deposition of undesired compounds on the membrane surface or inside the pores (i.e., fouling), leads to a permeability decline, requiring frequent cleanings (plant stoppage, greater water, and chemical consumption) and higher working pressures, thus increasing energy consumption. Besides, PA has a low tolerance to oxidising agents, especially to chlorine, which is commonly used as a biocide during the feed pre-treatment stage aiming at reducing biofouling in the membranes. Even if sodium bisulphite is added to eliminate the remaining free chlorine, frequently residual free chlorine concentrations reach the RO stage. Chlorine is bonded to the amide and to the aromatic groups in PA (i.e., N-halogenation), causing their chlorination and posterior hydrolysis by oxidation, thus reducing the rejection capacity of the membranes [54]. Accordingly, S.P. Nunes et al. [18] identified fouling and chlorine resistance as the main current challenges in the development of future long-lasting RO membranes for water treatment. 

It is widely recognised that high hydrophilicity, a smooth surface, and enhanced electrostatic repulsion of common foulants are directly correlated with enhanced resistance to fouling deposition [55,56]. Moreover, reducing N-halogenation reactions results in an upgraded resistance to chlorine. In this line, increasing the steric impedance for chlorine bonding, and the incorporation of chlorine-resistant advanced materials can upgrade PA resistance to chlorine attack [54]. To achieve such properties, different strategies can be followed, including the surface modification of commercially available TFC-PA-RO membranes and the synthesis of novel RO membranes.

Surface modification strategies have been widely researched and reviewed in the last few years [56,57,58]. Dip coating is one of the most used strategies for surface modification. Among other materials, mussel-inspired catecholamines have been comprehensively explored as biobased, water-soluble modifying materials to create adhesive, smooth and hydrophilic coatings [59,60,61,62]. Less frequently, dip coating has been used to incorporate advanced functional materials on mixed-matrix coatings (e.g., silver nanoparticles [63] and silane [54]), which have been demonstrated to improve both fouling and chlorine resistance. Otherwise, graft polymerisation has been employed to attach hydrophilic monomers to the membranes (e.g., biobased L-Lysine [64], hydroxyl methacrylate [65]) and to polymerise them as side chains. Additionally, the surface grafting of sacrificial regenerative layers (e.g., glycylglycine) has been proposed as a strategy to consume active chlorine by their N-H pendants or via chlorine oxidation, enhancing the service time of the PA layer [66]. Layer-by-layer has been reported as a versatile strategy to create nanostructured coatings on the membranes, incorporating either charged hydrophilic polyelectrolytes (e.g., polyethyleneimine [67]) or advanced chlorine-resistant functional materials (e.g., graphene oxide [68], titania nanosheets [67]). Otherwise, casting biobased materials such as chitosan and polyvinyl alcohol can enhance the antifouling properties of new and used membranes, in an attempt to renew their surface [69]. However, the incorporation of additional coating layers on the membranes often results in a permeability decline [70,71]. Plasma modification is another strategy enabling the modification of the outmost membrane surface to introduce desired functionalities by its exposure to a reactive environment. As long as no additional coating layers are required to improve surface hydrophilicity, plasma-modified membranes usually show enhanced permeability [72]. 

Differently, the synthesis of new-generation RO membranes facilitates the incorporation of advanced nanomaterials during the interfacial polymerisation step, creating mixed matrix membranes. The preparation of mixed matrix membranes avoids assembling additional membrane coatings, thus facilitating keeping or even improving water permeability. In addition, avoiding additional membrane modification steps would considerably reduce the costs associated with manufacturing novel antifouling membranes, as current industrial membrane manufacturing lines could still be usable. For instance, the incorporation of graphene-oxide nanosheets [73], multi-walled carbon nanotubes [74], and titanium dioxide nanoparticles [75] has been demonstrated to simultaneously enhance fouling and chlorine resistance, resulting in improved durability. Furthermore, these studies reported a higher permeability and rejection capacity of the novel membranes. 

Moreover, replacing the active layer of PA with an active layer of PES resulted in outstanding chlorine resistance properties in the PES TFC RO membrane in comparison with a commercial PA SWRO membrane (i.e., the salt rejection was stable after 100,000 ppm h ClO^−^ in the case of PES-RO, whereas it was considerably degraded after 8000 ppm h in the case of PA-RO). The PES TFC RO membranes were fabricated by the layer-by-layer interfacial polymerisation of 3,5-dihydroxybenzoic acid with trimesoyl chloride. The ester linkages in the PES active layer and the steric hindrance of 3,5-dihydroxybenzoic acid, among others, could contribute to the greater resistance to chlorine in the PES TFC RO membrane [76].

Aside from membrane properties, feed spacers also have a considerable effect on the fouling deposition rate. An optimal feed spacer design should increase the turbulence of the feed to reduce fouling and concentration polarisation phenomena without a significant increase in pressure losses [77]. In this sense, it has been demonstrated that three layers spacers (instead of conventional two layers) [78], 3D printed sinusoidal spacers [79], and spacers with helical filaments [80] improve the local feed turbulence and thus can mitigate (bio)fouling. 

Despite surface modification and the preparation of novel RO membranes having been largely studied at a laboratory scale, there are still several issues that remain unsolved, such as addressing the scalability, long-term stability, and cost analysis of those novel manufacturing strategies, which insights into the existence of a consistent gap between academic effort and real industrial needs. 

#### 2.2.3. Facilitated Reuse and Recycling

According to eco-design principles, membrane reuse and recycling strategies should be ensured at the design stage. Up to now, these strategies have been developed once the end-of-life element was generated. In this way, indirect recycling strategies have encountered common limitations, such as complicated module disassembling due to the current conventional fibreglass casing and the presence of plastic components with low recyclability (fibreglass, rubber, and glued parts). These facts are described in more detail in the corresponding section (Section 3.3). Therefore, apart from biobased membrane engineering, future studies should address the eco-design of RO modules, to facilitate RO module disassembling and improve the recyclability of the RO module components. Indeed, as depicted in Figure 2, the RO module components account for the major proportion of the weight of the RO module (i.e., 58% of the weight of the RO module). For instance, 3D printing using biopolymers, such as polylactic acid, cellulose, and polybutylene succinate, could be a future strategy to enhance the sustainability of the spacers and end caps and permeate the tube of the module [81].

### 2.3. Fouling Prevention and Mitigation during the Usage Phase

Proper maintenance during the usage phase can help to extend membrane service time by preventing and mitigating membrane fouling. The next sections summarise conventional and advanced maintenance methods. 

#### 2.3.1. Feed Pre-Treatment Technologies

Feed pre-treatment allows for the elimination of the main foulants, effectively preventing fouling occurrence in RO membranes. Conventional pre-treatment methods include chemical processes (e.g., the addition of antiscalants and dispersants, coagulation and electrocoagulation, followed by flocculation, chlorination, ozonisation) and physical processes (e.g., media filtration and dissolved air filtration) [82]. Usually, a chemical pre-treatment such as coagulation/flocculation is followed by a physical pre-treatment such as media filtration or dissolved air filtration to eliminate the added chemical. For instance, the complete elimination of chlorine is crucial to avoid PA degradation in RO membranes. In this sense, innovative pre-treatment methods such as nano photocatalytic and nano silver addition have been recently identified at a laboratory scale as promising pre-treatment methods avoiding the use of PA-degrading oxidants (e.g., chlorine and ozone) [83]. A recent study underlines the importance of the elimination of siliceous foulants in municipal potable reuse RO plants, as it was found that this type of foulant was the major cause of irreversible fouling and performance loss of the membranes [84].

At a large scale, membrane-based pre-treatment methods using MF, UF, or NF membranes are cost-effective and have high performance in reducing membrane replacement at the RO stage. Furthermore, membrane-based pre-treatment methods require a lower amount of chemicals than previous examples [9,82,85]. Moreover, recycled NF and UF-like membranes have formerly demonstrated high applicability for efficiently removing the main foulants from the feed [86], which further increases the value chain of desalination membranes, as it is detailed in Section 3.2.

#### 2.3.2. Early Fouling Detection Methods

The real-time monitoring of the fouling potential of the feed and the state of the membranes can help to predict and to detect fouling early, being able to set membrane cleaning steps accordingly. The characterisation of the feed (physicochemical parameters, organic content, and bacterial quantification) can serve to evaluate its fouling propensity and support consequently the optimisation of feed pre-treatment methods [87]. For instance, the real-time measuring of the inorganic salt content (e.g., calcium sulphate, calcium carbonate, calcium phosphate, etc.) and pH of the feed water enables an efficient addition of antiscalants products [88]. An innovative approach is the development of a smart, real-time optimisation algorithm to avoid under- and overdosing for a more efficient reduction in the scaling at RO membranes [89]. Modified Fouling Index Ultrafiltration (MFI-UF) has been reported as a significant development to assess the particular and colloidal fouling potential of feed water, by a standard UF testing procedure [90]. Regarding the biofouling potential, Assimilable Organic Carbon (AOC) and Bacterial growth potential (BGP) are key parameters to evaluate the microbial growth potential of the feed water based on its carbon content (e.g., acetate) and biodegradable organic matter content, respectively [91]. 

Fouling real-time monitoring can be performed either in situ, in the membrane cascade, or ex situ, in a side stream or “canary cell”, reproducing the conditions of the RO plant [87]. In RO plants, the state of the membranes is generally predicted by monitoring pressure drops, transmembrane pressure, and rejection coefficients [92]. Nevertheless, advanced fouling detection methods (e.g., ultrasonic, optical, and electrical methods) allow for the online monitoring of the membrane state through more precise, non-invasive characterisation techniques. For instance, ultrasonic time domain reflectometry employs ultrasonic waves to characterise perturbations in the media (i.e., frequency and bandwidth of the wave), which can be analysed for monitoring scaling and membrane cleaning performance [93]. Two-dimensional fluorescence spectroscopy has been proposed as an optical method allowing for online monitoring of organic and biofouling in membrane-based processes [94]. Two-dimensional fluorescence spectroscopy provides the fluorescence excitation-emission matrices (EEMS) of the membranes, containing information about a complex biological media. Regarding electrical methods, electrical impedance spectroscopy has been validated for the ex situ monitoring of fouling and cleaning efficiency in a large-scale water treatment plant, being able to identify the nature of fouling by observing the variations in the electrical impedance [92]. 

#### 2.3.3. Membrane Cleaning

Despite the improvements in fouling resistance and the implementation of feed pre-treatment and early fouling detection methods, at last, fouling remains inevitable [95]. Cleaning methods are then applied for the reduction in fouling based on the type of fouling to be removed, achieving a partial recovery of the initial hydraulic permeability. Conventional physical cleaning treatments include flushing and backwashing with pressurised water, sponge ball washing, CO_2_ permeation, and osmotic backwashing [85]. Other advanced physical cleaning treatments have been developed based on ultrasonic [96] and electromagnetic fields [97]. Regarding chemical cleaning, acids (e.g., hydrochloric acid, sulfuric acid, citric acid) are commonly used for the elimination of scaling, while alkali (e.g., sodium hydroxide), chelating agents (e.g., ethylene diamine tetra acetic acid, EDTA), and surfactants (e.g., sodium dodecyl sulphate) are employed for the elimination of organic fouling, and lastly, biocides (e.g., sodium bisulphite) are used for the elimination of biofouling [98]. RO membrane cleaning enables the partial recovery of membrane permeability, extending their service time. However, the application of frequent cleaning stages requires the stoppage of the plant, depleting consequently its productivity and resulting inevitably in the generation of a significant volume of chemical waste [85]. In addition, chemical cleaning could accelerate the deterioration of the selective PA layer, compromising its rejection capacity and, therefore, its durability [85].

## 3. End-of-Life Membrane Management 

Membrane fouling and performance deterioration are up to now inevitable drawbacks accompanying membrane technology. When performance decays, membranes are replaced, generating an increasing amount of waste. Currently, landfill disposal is the most frequent fate of EoL RO membranes [9,99,100]. Landfilling generates greenhouse emissions, toxic leachates, odours, and visual impacts [101,102]. Moreover, it implies the loss of valuable raw materials and energy [103]. Likewise, the transport of the EoL membranes to landfill facilities results in significant greenhouse emissions [8]. 

To tackle this unsustainable situation, the waste management hierarchy should be urgently implemented. As a consequence, membrane reuse, recycling, and recovering alternatives are gaining increasing attention from the research and industrial stakeholders [9,53,95,104].

**Figure 4:**
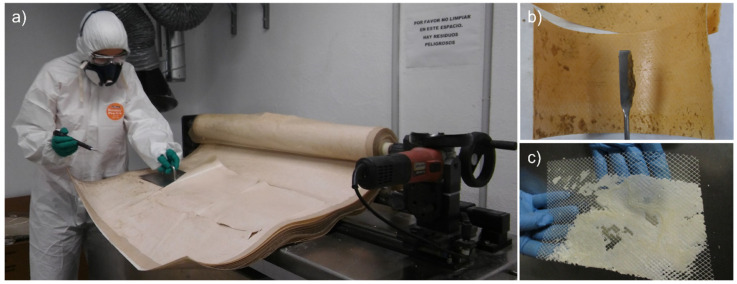
(**a**) Opened RO module and extraction of membrane and spacer samples. Reproduced with permission from Ref. [105]. Copyright 2022, A. Lejarazu-Larrañaga et al., (**b**) taking membrane fouling samples for analysis (presumably organic fouling), (**c**) a sample of a turbulence promoter (feed spacer) with scaling deposits.

To adopt rationally a competent membrane valorisation strategy, EoL membranes are characterised in terms of module weight, permeability, rejection coefficients, and fouling degree and nature. The characterisation of a single RO membrane module can contribute to estimating the state and the cause of the failure of a batch of membranes, operating under similar conditions during their service time. Complementarily to the characterisation of the main membrane performance-determining parameters (i.e., hydraulic permeability and salt rejection coefficients), the deconstruction of the original spiral wound module configuration (module autopsy) enables a deeper analysis of the fouling degree and nature, helping to disclose the main causes of the performance deterioration or failure (Figure 4). 

**Figure 5:**
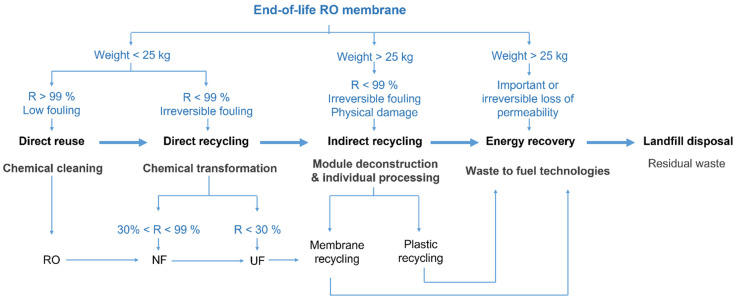
Cascade open loop reuse, recycling, and recovery alternatives for EoL RO membrane modules. R, Rejection coefficient; RO, reverse osmosis; NF, nanofiltration; UF, ultrafiltration.

The deterioration of RO rejection coefficients (to *R* < 99%) could impede their direct reuse as RO membranes, while membranes presenting a high amount of scaling (>25 kg of the weight of the EoL module, considering that the original average weight of a pristine 8″ RO module is between 11 and 16 kg [8]) might not be suitable to any reuse or direct recycling processes, but could still be processed following an indirect recycling strategy. Figure 5 shows EoL RO module management alternatives according to the conditions of the modules at the end of their service time [95]. Complementarily, to help in the decision-making process, MemEOL [106] and REMapp [107] interactive online free tools were created through the collaboration of several research groups and experts working in the field of membrane reuse and recycling. 

As depicted in Figure 5, membrane reuse, direct and indirect recycling, and recovering alternatives could be complementarily implemented in a cascade mode, thus bringing to the EoL elements several additional life cycles, extending considerably their lifespan and improving their value chain. These cascade valorisation technologies could enable the implementation of EoL membranes and components in different applications from their original purpose (i.e., open loop recycling) [108], as it is analysed in the following sections. 

### 3.1. Direct Reuse

**Figure 6:**
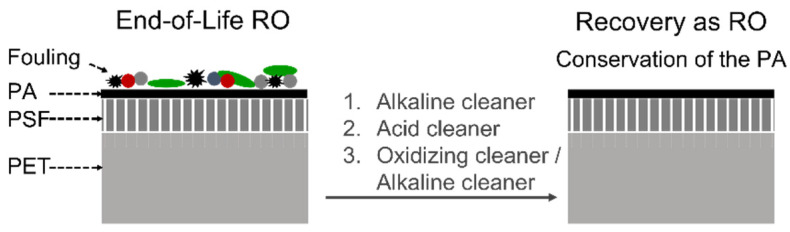
Schematic illustration of EoL RO membrane recovery for direct reuse as RO. PA, polyamide; PSF, polysulfone; PET, polyester.

In the case of EoL membranes presenting high salt rejection coefficients (*R* > 99%), a direct reuse strategy should be prioritised. Direct reuse aims to recover the RO performance (in terms of permeability and salt rejection capacity) by a chemical cleaning, without the intended degradation of the selective PA layer. Previous studies [109] proposed a chemical cleaning based on the use of an alkaline cleaner (i.e., a mix of NaOH, phosphate surfactants, and sequesters), followed by an acid cleaner (i.e., a mix of HCl and H_3_PO_4_). In some cases, a third step might be applied using either oxidising agents (i.e., H_2_O_2_) or a second round of the alkaline treatment (Figure 6). 

Those treatments resulted in some alteration of the RO salt rejection capacity (*R* varied in the range between −29% and 8%), along with the enhancement of the membrane permeability (from ~3 L m^−2^ h^−1^ in new brand RO elements up to 261 L m^−2^ h^−1^ after the cleaning treatment). The reusability of RO membranes has been validated at a pilot scale in several applications of a lower water quality requirement, including desalination as “sacrifice” membranes (i.e., in the first or the last positions of a pressure tube, where the effects of fouling and scaling, respectively, are more pronounced) [109], reclaimed water production in tertiary wastewater treatment [109], and landfill leachate treatment [110].

### 3.2. Direct Recycling 

A direct recycling strategy should be prioritised in the cases of EoL membranes with a degraded salt rejection capacity (*R* < 99%), but likely presenting a relatively low amount of fouling (weight < 25 kg), and in the case of spent reused membranes presenting the abovementioned conditions. 

Direct recycling aims to modify the filtration properties of EoL RO membranes to obtain membranes with nanofiltration (NF) and ultrafiltration (UF) properties in terms of the salt rejection capacity and hydraulic permeability while maintaining the spiral wound configuration. PA-TFC RO membrane recycling methodologies have been developed based on the low tolerance of the PA to exposure to oxidising agents. Since the first studies were conducted in the early 2000s [111,112], different oxidising agents have been tested, including H_2_O_2_, NaOH, KMnO_4_, and NaClO [21,99,113,114,115,116]. Among them, NaClO treatment achieved the greatest permeability in recycled membranes [21]. In addition, NaClO solution presented better chemical stability and reusability, allowing for the production of a lower volume of effluents in a large-scale application [114] and, therefore, it was used in further studies [86,99,115,116]. Regarding the scaling up of the direct recycling process, the comparison between the passive immersion of the membranes in the NaClO solution versus the active recirculation of the NaClO solution through the membrane by a pumping system was assessed in [117]. In this study, the cost-effectiveness of the passive recycling systems was highlighted. 

**Figure 7:**
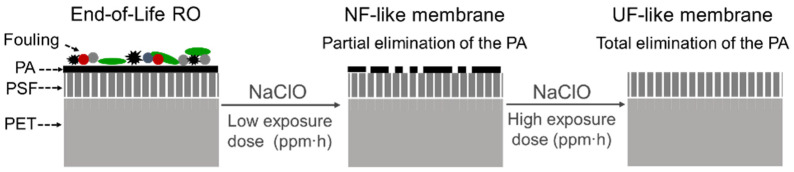
Schematic illustration of EoL RO membrane direct recycling by transformation to NF and UF-like membranes. PA, polyamide; PSF, polysulfone; PET, polyester. Adapted with permission from Ref. [86]. Copyright 2018, R. García-Pacheco et al.

The exposure to a concentrated free chlorine solution eliminates membrane fouling, while the dense PA layer is intentionally degraded either partially, attaining NF properties, or totally, attaining UF properties (see Figure 7 and Table 1).

**Figure 8:**
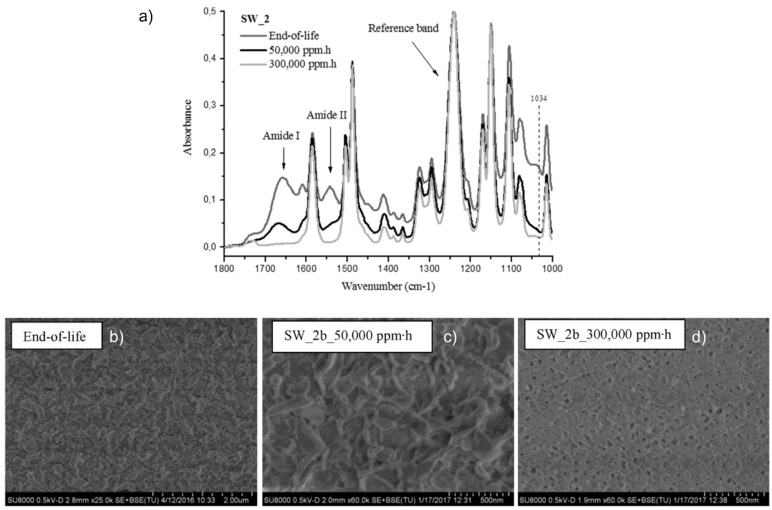
Surface characterisation by (**a**) ATR-FTIR of EoL RO SWRO membrane and recycled NF and UF-like membranes (50,000 ppm h and 300,000 ppm h exposure doses to NaClO, respectively). Surface morphology by SEM, (**b**) EoL SWRO membrane, (**c**) recycled NF-like membrane with remaining PA (after 50,000 ppm h exposure dose to NaClO; and (**d**) recycled UF-like membrane with the porous PSF surface (after an exposure dose of 300,000 ppm h to NaClO). Reproduced with permission from Ref. [119]. Copyright 2018, S. Molina et al.

Membrane characterisation is essential to ensure the achievement of UF and NF properties. On the one hand, membrane performance is commonly characterised in terms of hydraulic permeability and salt rejection capacity by filtering synthetic brackish water solution (i.e., an aqueous solution of NaCl, MgSO_4_, and dextrose) [99]. On the other hand, the elimination of the PA layer is frequently confirmed by surface characterisation. Accordingly, Attenuated Total Reflection-Fourier Transform Infrared (ATR-FTIR) spectroscopy is frequently used to analyse the partial or total depletion of amide I and II peaks in the recycled NF- and UF-like membranes, respectively (Figure 8a). Scanning Electron Microscopy (SEM) is commonly employed to detect the remaining PA in NF-like recycled membranes (Figure 8b) and to confirm its complete elimination by identifying the nano-porous polysulfone surface in the recycled UF-like membranes (Figure 8c) [119]. 

The exposure dose needed to reach NF- and UF-like properties depends on several factors, including (i) the initial conditions of the RO membrane (i.e., the % salt rejection and hydraulic permeability of the EoL membrane); (ii) the type of RO membrane (designed for SWRO or BWRO); and (iii) storage conditions (i.e., dry-stored membranes need to be pre-treatment with an aqueous ethanol solution for the rewetting of the pores) [115]. In general terms, highly damaged membranes and SWRO membranes require a higher exposure dose to the oxidising agent for the elimination of the PA layer [99]. As a result, the standardisation of the exposure doses needed to reach NF and UF properties is complex. Roughly, the exposure doses can be ranged between 1000–150,000 ppm h to reach NF performance, and 10,000–400,000 ppm h to achieve UF performance [120].

The performance of recycled NF and UF membranes has been evaluated at the laboratory and pilot scales, validating the recycled membranes for various applications, including, (i) as the pre-treatment of the feed before the RO stage [86]; (ii) in natural brackish water desalination to produce water of a lower quality requirement (i.e., for irrigation) [86,121]; (iii) as fusible or sacrifice membranes in desalination (covering the positions of the RO pressure vessel where fouling is more frequent) [86], (iv) as tertiary treatment in wastewater reclamation [110,111,112], (v) for the production of safe drinking water in a household gravity-driven water treatment system [122], and (vi) in the clarification of wet-process phosphoric acid from rock phosphate [123].

### 3.3. Indirect Recycling

When membranes present an assumably high amount of scaling (EoL weight > 25 kg) and in the case of spent recycled membranes, the indirect recycling approach enables the individual management of flat sheet membranes and other module components by disassembling the RO module. The first indirect membrane recycling studies were conducted in 2019 [124]. Accordingly, among other EoL RO membrane valorisation strategies, these are the most recently developed alternatives.

#### 3.3.1. Recycling Flat Sheet Membranes

**Figure 9:**
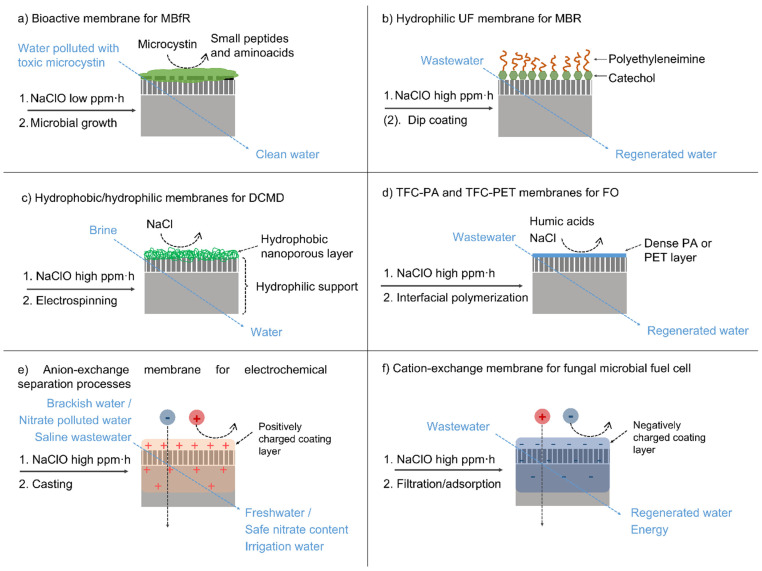
Schematic representation of innovative indirect EoL RO membrane recycling approaches.

Obtaining the flat sheet membranes from the EoL RO module increases markedly the versatility of applicable membrane recycling and modification treatments, with respect to a close, spiral wound module configuration. Thus, the applications of indirectly recycled membranes are considerably broadened. Before applying a modification layer, membranes are commonly pre-treated using NaClO at different exposure doses depending on the modification requirements, eventually removing the fouling and the PA layer. Up to date, indirectly recycled EoL RO membranes have been validated at a laboratory scale in membrane biofilm reactors, bioreactors, membrane distillation, forward osmosis, and electrochemical processes, as depicted in Figure 9 and detailed below.

Based on the inherent capacity of bacteria to become attached to the membranes and to grow on a biofilm, J. Morón-López et al. [125] cultivated a microcystin degrading biofilm on the surface of the discarded membranes (after NaClO pre-treatment), obtaining low-cost eco-friendly biological filters for a membrane biofilm reactor (MBfR) system (Figure 9a). In this case, a low exposure dose to NaClO resulted in a greater adhesion of the biofilm to the membrane [126], while air supply during the bacterial colonisation phase enhanced the biofilm formation and microcystin degrading capacity [127]. The bioactive membranes were employed in MBfR for microcystin removal from polluted water, attaining a removal of 2 ppm microcystin within 24 h. In addition, this concept was demonstrated to be economically competitive with respect to conventional treatments for microcystin removal [124].

Considering the similar filtration properties of recycled UF membranes to conventionally used membranes in membrane bioreactors (MBRs), L. Rodríguez-Sáez et al. [128] implemented recycled flat sheet UF-like membranes (i.e., after a high exposure dose to NaClO) in a plate and frame configuration aerobic MBR for wastewater treatment. The recycled UF membranes showed a similar performance to the commercial MF membrane in terms of permeate flux and rejection and a greater fouling resistance, which might be attributed to the presence of residual chlorine in the recycled membranes. A compelling advantage of this approach is the possibility of using recycled flat sheet UF-like membranes without further modification. A different study [129] addressed the surface modification of the recycled membranes using biobased compounds (catechol and polyethyleneimine), by which the antifouling properties of the recycled membranes were further enhanced (Figure 9b). 

Otherwise, adding a new selective layer to flat sheet discarded membranes could result in outstanding rejection capacities. Accordingly, J. Contreras et al. added trough electrospinning of a polyvinylidene fluoride, a thin nanofibrous hydrophobic layer, to UF-like membranes, attaining a 99.99% rejection factor in brine desalination under the direct contact membrane distillation (DCMD) process (Figure 9c). Brine desalination represents an important pathway to achieve zero liquid discharge in desalination. In addition, concentrated brines facilitate brine mining, aiming at recovering valuable elements and compounds present in seawater (e.g., lithium). 

In a distinct study, a dense polymeric layer of either PA or PET was deposited on the PSF surface of recycled UF-like membranes by interfacial polymerisation. The recycled membranes were validated for saline wastewater treatment by forward osmosis (FO) technology, achieving a high rejection of humic acids and salt, which was even superior to the commercial FO membrane in the case of the recycled membrane incorporating the new PA layer. Moreover, the morphological and structural characteristics of the recycled membranes were found to be similar to those of commercial FO membranes, indicating their suitability for FO applications (Figure 9d) [130]. 

The introduction of a high number of fixed charged functional groups in membranes could result in considerable Donnan repulsion forces, producing highly permselective ion-exchange membranes. In this line, A. Lejarazu-Larrañaga et al. deposited a new charged coating layer on the flat sheet UF-like membranes by casting a polymeric mixture containing a finely grounded anion-exchange resin (Figure 9e). The resulting membranes showed a high permselectivity (87%) and excellent mechanical properties, inherited from the RO membrane [131]. Although the electrical resistance was found to be relatively high (77 Ω·cm^2^), it was significantly reduced by an acid-alkali activation treatment, resulting in the improved performance of the membranes in brackish water desalination by electrodialysis (ED) [132]. Moreover, the applications of the recycled anion-exchange membranes could be broadened by providing an enhanced selectivity towards specific ions, such as nitrate, which could facilitate its selective recovery from wastewater. In this line, nitrate selective properties were achieved in [133] by the incorporation of a hydrophobic ion-exchange resin in the membrane attaining a higher rejection of highly hydrated ions, such as sulphate, and increasing the transport of less hydrated ions, such as nitrate. Moreover, passive electrochemical transport processes, such as Donnan dialysis and the ion-exchange membrane bioreactor, were studied for the elimination of nitrate from polluted water. Such processes could enable water decontamination at a minimum energy requirement by the implementation of low-cost recycled membranes as anion-exchange membranes (i.e., 2.4 EUR m^2^ for the cost of the raw materials used in membrane preparation) [105]. In a very recent study, recycled anion-exchange membranes and recycled NF membranes have been employed for the treatment of saline synthetic urban wastewater, both reaching a suitable demineralisation rate of the treated water (i.e., conductivity and SAR value), enabling its reuse for crop irrigation [134]. Using a different approach, A. Somrani et al. [135] used a flat sheet discarded RO membrane, after a high exposure dose to NaClO, for the preparation of a cation-exchange membrane by the filtration/adsorption of a polystyrene sulfonic acid (PSS) electrolyte solution (Figure 9f). The resulting membranes were employed in a fungal microbial fuel cell, by which wastewater treatment and energy production processes were coupled and simultaneously attained. The MFC using the recycled membranes achieved a similar power density to the cell assembled using Nafion^®^ 117 membranes. In addition, the recycled membranes showed lower surface roughness in comparison with Nafion^®^ 117, which might result in better antifouling properties.

As mentioned, these technologies have been very recently developed; therefore, their implementation at a large scale has not been yet addressed. Future works should evaluate the long-term stability of the recycled membranes along with their respective life cycle and economic assessments. In that respect, recycling processes avoiding further modification treatments or additional modification layers would represent advantageous environmental and economic implications and thus facilitate the scaling up of the technology. For instance, after NaClO treatment, without adding further modification layers, the flat sheet UF-like membranes are ready to be assembled in a plate and frame MBR, reducing the environmental burdens and economic costs associated with the requirement of additional modification steps. Similarly, the development of biofilms for recycled membrane biofilm reactors is an environmentally friendly and low-cost modification approach. Lastly, alternative life cycles for the indirectly recycled membranes at the end of their service time should be proposed and studied. 

Altogether, indirect recycling shows a high potential for the valorisation of spent recycled membranes and highly damaged membranes, resulting in the production of recycled membranes for a broad number of applications and allowing for the individual recycling of plastic module components. Accordingly, further research in this direction could be expected in the upcoming years.

#### 3.3.2. Recycling Other Module Components

**Figure 10:**
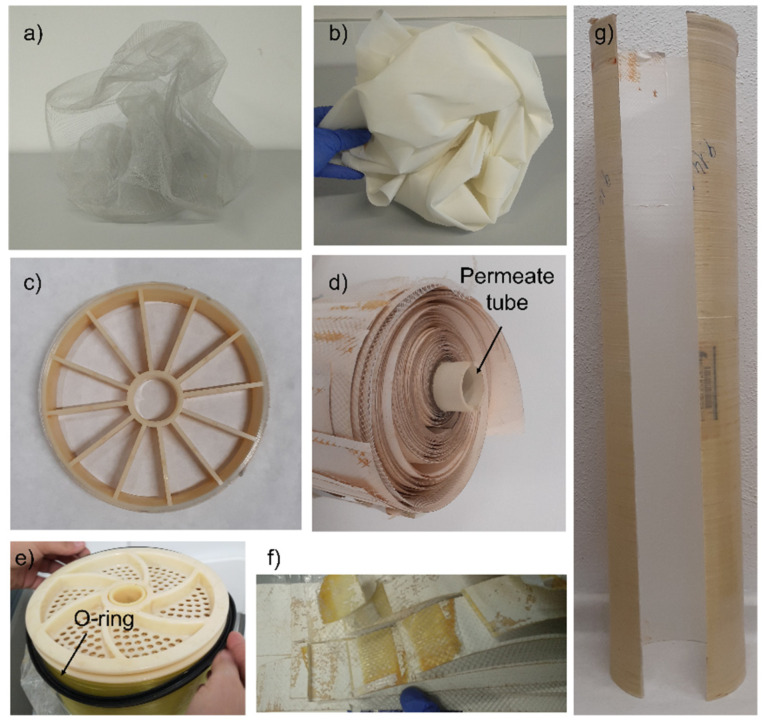
(**a**) PP feed spacer, (**b**) PET permeate spacer, (**c**) ABS end cap, (**d**) ABS permeate tube, (**e**) rubber o-ring, (**f**) glued parts, (**g**) fibreglass casing.

As mentioned before, apart from the membranes, the 58% of the weight of a conventional RO module belongs to distinct plastic materials (PP feed spacers, PET permeate spacers, ABS end caps and permeate tube, glued parts, fibreglass casing, and rubber) (Figure 10), which can be individually managed owing to the indirect recycling strategy.

The valorisation route will depend on the composition of each material. Three different main routes could be followed, direct valorisation, mechanical recycling, and chemical recycling, as it is summarised in Table 2. 

Direct valorisation strategies are those that do not require further processing of the components to be valorised and, thus, represent minimal environmental and economic burdens. For instance, feed and permeate spacers could be used directly as geotextile materials [116]. Non-woven geotextiles are widely used in road construction to provide mechanical reinforcement and in agriculture and gardening to prevent the mixture of different soil layers while providing proper water drainage, among others [147]. The current geotextile industry is a large user of petroleum-based polymers (>1.4 billion m^2^ geotextile produced per year and 98% of them are petroleum based) [147], and in this sense, the use of recycled materials would bring more sustainability to the sector under an open loop recycling approach. 

Moreover, as long as feed and permeate spacers are flat sheet components with consistent mechanical properties, their direct valorisation as supporting materials in the preparation of membranes should be investigated, especially that of the PET permeate spacers with a considerably closer structure. Accordingly, membranes for membrane distillation purposes were prepared by chemical modification in [137], following the methodology illustrated in Figure 9c and described previously, using both feed and permeate spacers as support. The resulting membranes achieved salt rejection coefficients as high as 99.75% and 99.99%, respectively. The lower rejection coefficient of the latter was attributed to the larger mesh size of the feed spacer. In the case of feed spacers, their direct valorisation as turbulence promoters in a plate and frame electrodialysis (ED) stack has been formerly proposed [131,132].

Apart from direct valorisation strategies, plastic waste recycling can be attained by either mechanical or chemical recycling. Mechanical recycling is currently the most common method for recycling plastic waste. In the case of thermoplastics, such as PP, PET, and ABS (representing 39% of the weight of the module), mechanical recycling routes are widely implemented on an industrial scale. The mechanical recycling of thermoplastics is enabled by sorting, shredding, melting, and extruding thermoplastics to form new products. For instance, in [131], the mechanical recycling of old feed spacers (PP) was tackled to extrude end caps and anolyte and catholyte compartments for a lab-scale ED stack, reaching 84% of recycled plastic content in the stack. 

In the case of composite thermosets (such as fibreglass and rubber), recyclability is more limited. Nevertheless, downsizing by shredding and granulation leads to powders that can be used as additive fillers in different applications [138,139,140]. For instance, fibreglass powder can be used as a reinforcing additive in many composites such as concrete, plastics, or roofing products, among others [138]. While in the case of powder rubber, its applicability as a filler in virgin rubber, concrete, or blended with polymers as a reinforcing filler has been reported [139,140]. 

However, mechanical recycling results in downgrading the polymer properties (e.g., mechanical strength), and, usually, recycled thermoplastics have to be blended with pristine plastics to improve their properties. As a result, there is a limited number of reprocessing cycles. To avoid such limitations, chemical recycling has emerged as a promising alternative for the valorisation of plastic waste. Chemical recycling is attained by depolymerisation under different processes, such as chemolysis, pyrolysis, gasification, and fluid catalytic cracking [108]. Such processes enable the conversion of the polymer into monomers and other by-products, which can be later used for the production of chemicals, fuels, or virgin plastics, with equal or superior performance to the original material [143].

Accordingly, the depolymerisation of PP by inductive coupled plasma (ionisation), results in a gas containing 94% of propylene [141], while PP recycling by oxidative thermolysis leads to the formation of acetic acid and other by-products (methanol, formic acid, and propionic acid) [142]. In the case of PET, glycolysis and aminolysis have been identified as the most promising chemical recycling methods to produce high-added value polymers, such as polyurethanes, polyisocyanurate foams, poly(ester amide)s, composites, and other materials [143]. In a screening of the chemical recycling of different polymers, J. Souza dos Passos et al. subjected ABS to subcritical hydrothermal liquefaction in an alkaline environment, obtaining an oil product composed of oligomers which required further upgrading to recover the chemicals of interest [144]. The chemical recycling of fibreglass was studied in [145]. In the refereed work, the authors aimed at recovering the glass fibres and the monomers of the resin by chemolysis using subcritical water as a solvent. However, the obtained results were not fully promising, as high process temperatures resulted in the degradation of both (recovered monomers and fibres), compromising the mechanical properties of the fibres and requiring additional purification steps. In the case of rubber, devulcanization by chemicals, thermos-mechanical, microwave, or ultrasounds converts old rubber into virgin material, which can be revulcanised to form new rubber, without degrading its properties [139,140]. Furthermore, chemical recycling by the H_2_O_2_-assisted hydrothermal method could allow for the valorisation of the complete RO module, avoiding membrane disassembling labours. A recent study [146], showed that pyrolysis at 600 °C of the complete RO module results in oil (28 wt%) and gas (18 wt%) fractions, which could be used as fuel and chemical feedstock, and a solid char fraction (22 wt%), which could potentially be used as carbon precursor for functional (N, S co-doped) carbon dots synthesis. Carbon dots are emerging nanomaterials with high potential for bioimaging, photocatalysis, and optoelectronics, among others.

Notwithstanding, chemical recycling methods are more complex and diverse in comparison with mechanical recycling and require in some cases a considerable amount of energy (e.g., thermolysis). As a consequence, these processes are not commonly implemented at an industrial scale yet [148]. 

Overall, the development of plastic waste valorisation strategies can alleviate the worldwide growing plastic waste pollution. Future studies should contribute to improving the efficiency of sorting the membrane components through a facile module disassembling, as well as addressing the recyclability of composite plastic blends. In this sense, the upcoming development of innovative chemical recycling processes could greatly contribute to increasing the proportion of polymeric waste suitable for recycling [149]. 

Lastly, glued parts, representing the non-recyclable fraction of the RO module (7% of the total module weight) could be fed into mixed plastic waste to fuel energy recovery technologies (Section 3.4) or could be disposed of in landfills as residual waste.

### 3.4. Energy Recovery

At present, the energy sector is in a serious crisis. Fossil fuel reserves are diminishing, while geopolitical tensions between producers and consumers are increasing, resulting in a historical raise of energy prizes, and consequently outcoming an imperative and urgent necessity to reach a resilient, energy self-sufficient economy. In this regard, waste-to-fuel technologies can importantly contribute to the production of fuels from abundant and local waste sources, reducing the volume of waste, while advancing towards energy self-sufficiency. 

Petroleum-derived plastics have an inherent high hydrocarbon content and thus present potential suitability as feedstock for fuel production. In particular, the carbon content of RO membranes and module materials is ranged between 30% and 88%, being fibreglass casing the component presenting the lowest carbon content (30–50%) [21]. Accordingly, energy recovery is considered a suitable practice for membranes that are deeply damaged (e.g., EoL module > 25 kg weight, and membranes presenting an irreversible loss of permeability) for spent recycled membranes, for the non-recyclable fraction of disassembled module components, and for spent recycled plastic components (Figure 5) [21,95]. Currently, the most employed technologies for energy recovery from solid plastic waste include incineration, pyrolysis, and gasification [150]. 

The first attempt to analyse the combustion of EoL RO materials was performed by C. Prince et al. [136]. They conducted a thermo-gravimetric analysis, attaining a 93% mass reduction in EoL RO materials under combustion above 900 °C. They found that fibreglass casing was the material producing the highest amount of residue due to its inorganic fraction (glass), comprised mainly of silica. As a result, they envisaged a high potential of EoL RO materials as feedstock in incineration, although they recommend addressing the emissions of such process carefully. Incineration is the most mature waste-to-fuel technology, whereas the emission of dioxins and furans and the production of toxic ash represent serious challenges to its sustainability. Accordingly, W. Lawler et al. [21] introduced pyrolysis and gasification as cleaner waste-to-fuel technologies with respect to incineration, and alternatively proposed the use of RO materials as coke substituents in the electric arc furnace steel-making process. Pyrolysis and gasification technologies offer similar power production capacities to incineration (between 104–1294 kWh ton^−1^ and 220–1730 kWh ton^−1^, respectively) while avoiding considerably the release of toxic hazardous emissions [151]. Owing to the elemental composition of the module materials, M Pontié et al. [152] calculated a heating value of 26 MJ kg^−1^ for TFC membrane sheets with a 61% carbon content, rising to 47 MJ kg^−1^ in the case of PP feed spacers composed of 85% carbon. In the case of the membrane sheets, their relatively high oxygen content (32%) reduced the calorific value, while their 2% sulphur would require the post-treatment of fuel. Still, membrane sheets and PP spacers showed higher heating values than most biomass fuels. Subsequently, it was estimated that the feed spacers in one ton of EoL RO modules could generate 2210 kWh of liquid and gas fuels. 

Overall, EoL RO materials have demonstrated a high potential for their use as feedstock in waste-to-fuel processes, allowing for a considerable reduction in the volume of waste and the recovery of the energy contained in polymeric materials with inherent high carbon content. Notwithstanding, such processes should be carefully evaluated in order to avoid emissions to air, soil, surface water, and groundwater, as well as risks to human health [153,154]. 

## 4. Life Cycle Thinking and Life Cycle Assessment 

The Life Cycle Assessment (LCA) and other related methodologies such as Life Cycle Cost Analysis and social-LCA enable a holistic evaluation of the sustainability of technologies, considering environmental, economic, and social dimensions. The LCA and related methodologies implement the life cycle perspective (i.e., from cradle-to-grave analysis), thus including all the life cycle stages, from the design and manufacturing to EoL management. In such a way, the identification and quantification of the potential environmental impacts and footprints are enabled using several categories as indicators, such as climate change, ecotoxicity, or resource depletion [22]. The standardisation of the LCA methodology allows for the comparison of the sustainability of different technologies. There are three main stages in an LCA: (i) goal and scope definition, (ii) the Life Cycle Inventory, in which the input and output flows to the biosphere and the technosphere (human/industrial system) are quantified, and (iii) the Life Cycle Impact Assessment, which characterises the inventory into impacts across the selected categories.

In the specific context of membrane technology, most of the analyses have been focused on the usage phase, analysing the implementation of the membranes in applications such as desalination or wastewater treatment [155,156,157,158,159], whereas, a lower number of studies have been devoted to the analysis of the EoL membrane management stage. However, the directive 2008/98/EC on waste already included the life cycle perspective and the Life Cycle Thinking considerations within the principle of extended responsibility of waste management from the side of manufacturers [11]. The first assessments of EoL RO management technologies were performed by W. Lawler et al. [100]. They screened the environmental footprint of several technologies, such as reuse, direct recycling into UF, plastic recycling, energy valorisation, and landfilling. At that moment, alternatives to landfilling for EoL RO membranes were developed at a low (1–3) Technology Readiness Level (TRL). As a result, the different waste management technologies were arranged in priority order according to their environmental outcome. These results were aligned with the waste hierarchy abovementioned. However, some considerations taken, such as the distance from membrane users (desalination plants) to a prospective recycling plant, as well as the lifespan of the recycled products, could have a significant impact on the final results, obtaining an unfavourable environmental balance for recycling alternatives. In such a way, it was estimated that a short lifespan of a recycled UF membrane along with a long distance between the end-user (desalination plant) and the recycling facilities could generate higher impacts than landfilling or incineration. 

The next LCA arrived together with the scaling-up of direct recycling into NF and UF [117], where Senán-Salinas et al. compared two membrane recycling pilots with different designs and introduced more detailed inventories of the recycling processes. One of the main aspects introduced was the variability of recycled membranes found in terms of resultant permeability and its dependence on the original design of the modules (i.e., designed to treat sea or brackish water). Therefore, the permeability difference factor was incorporated into the analysis. In addition, indicators such as the minimum lifespan were estimated through LCA to identify the thresholds. For instance, it was determined that recycled modules into NF or UF from BWRO have to ensure a lifespan of over 0.7 years to guarantee a favourable environmental outcome of the recycling process. This short lifespan threshold is possible due to the relatively low impact of the recycling process (1.95–2.5 kg CO_2_-eq per module) concerning the high impact of the production of new membranes (NF: 92.5 and UF: 188 kg CO_2_-eq per module). Moreover, the selected recycling pilot allowed a cost-effective process, with an estimated cost of 29.9–41.53 EUR per module.

Apart from the pilot analysis, the transport influence on the supply chain of the recycled membranes was also assessed in a posterior work that projected a recycling plant in Spain coupled with Geographic Information Systems (GIS) [8]. This work compared the whole supply chain of recycled membranes with newly produced counterparts, considering reverse logistics, the distribution of recycled and new produced modules, waste characterisation, and the selection of the EoL RO supply chain. The inclusion of complete supply chains evidenced a higher impact than estimated in previous works, ranging between 3.63–4.82 kg CO_2_-eq per module in the case of national distribution and reaching 13.3 kg CO_2_-eq per module in international distribution. However, it was shown that the environmental benefit of the recycling value chain was significant when comparing it with production and the distribution chains of new membrane modules, obtaining an avoided impact of 98.5 to 199 kg CO_2_-eq per module. As a consequence, the required lifespan ratios for the recycled membranes were higher than in previous works. Regarding the economic dimension, the minimum prices for cost recovery were calculated to be between 100–150 EUR per recycled module. In addition, the referred work [8] proposed the use of classification machine learning models for the rapid characterisation of EoL-RO modules and the identification of the optimal management route, thus referring to the high potential applicability of such technology to prospective membrane recycling processes. Machine learning and artificial intelligence are meant to solve common problems in process implementation through meaningful predictions based on data analysis by the use of computer systems, algorithms, and statistical models, and could represent a great advance for process-optimisation discipline [160]. Regarding indirect recycling strategies, the application of LCA to these technologies is still very scarce. This is understandable, considering that these approaches have been very recently developed. To date, only one case of LCA study can be found in the literature: the indirect recycling into forward osmosis membranes [161]. The most important conclusion of this work is the potential benefits gained when recycling module plastic materials, which was observed as a great complement that increases the environmental benefits of indirect membrane recycling. These recyclable parts included PP and PET spacers, ABS permeate tubes, and the end caps. This study also concluded that the relevance of membrane recycling in the environmental benefits depends on the amount of membrane recovered (69%) and the counterpart analysed (Cellulose Triacetate or TFC FO membranes) due to the different solvents used in their preparation. In a general conclusion, the authors concluded that the studied indirect recycling methodology should be placed in the management hierarchy after the direct recycling into NF and UF and before plastic recycling. Furthermore, in the referred work, the mass balances of the presented semiconservative open-loop recycling alternative were included based on the results of a previous study (i.e., a close-loop RO membrane recycling study) [100]. It is remarkable to mention that there are very few studies on membrane recycling reporting closed mass balances. In this sense, further assessments of plastic recyclability and close-loop recycling should be performed to assess more rigorously the mass percentage of the plastic that undergoes to recycled and the quality of the recycled plastic and thus the environmental credits offset.

As mentioned before, reuse, recycling, and recovering strategies could be complementarily implemented within an open loop cascade [128]. Nevertheless, fibreglass casing is a critical point which could hinder the implementation of indirect recycling strategies as a consequence of difficulties with dismantling the RO module. Therefore, the assessment of alternative materials for the substitution of conventional fibreglass should be addressed in the future. From the economic point of view, compared to direct recycling strategies, opening the membrane module during the indirect recycling process demands an important amount of labour, which would increase the cost of recycling. In addition, it has to be taken into account that a part of the membrane area can be not recovered when adapting the membrane from the spiral wound configuration to a plate and frame configuration. In the case of the plate and frame UF-MBR, it was estimated that using different frames allows for the recovery of 39 to 70% of the original membrane area [128]. Nonetheless, the cost of recycling was estimated to be in a competitive range (r-MBR: 5.91–10.56 EUR·m^−2^ and commercial-MBR: 12.38–20.63 EUR·m^−2^). These results demonstrated that, even with the actual limitation of a complicated module disassembling and a partial membrane area recovery, indirect recycling can still be a cost-effective alternative. Additionally, the authors proposed improvements in the future design for a facile deconstruction in order to decrease the cost of recycling (i.e., eco-design).

Lastly, it is important to ensure the transparency and reproducibility of the LCA and LCC studies. In this sense, the publication of the inventories used for the estimations in open data access journals or repositories is essential to contribute to the reliability of the published results [162,163]. However, the exportability of the models is limited in most cases by the software used for the modelling, and thus further efforts should be attempted in publishing models in other repositories such as GitHub. 

## 5. Future Outlook and Perspectives 

The integration of an eco-design perspective at the manufacturing stage, correct membrane maintenance during usage, and the implementation of EoL reuse, recycling, and recovering alternatives are essential to reduce the concerning waste generation in the RO desalination industry. Excluding membrane maintenance strategies at the usage phase, eco-design in manufacturing and EoL alternative management options are yet to be implemented at an industrial scale. In this line, the encountered limitations and future research directions could be addressed as follows:

- Biopolymers, recycled materials, and green solvents have an essential role in future sustainable polymer and membrane science. Accordingly, research in those fields is rapidly growing. Even if a considerable number of works have been devoted to the preparation of biobased membranes at a laboratory scale, the larger-scale implementation of biobased membranes, long-term studies, and the LCA of the technologies are yet to be explored. In addition, most of the literature on biobased membrane manufacturing is focused on MF, UF, NF, or pervaporation membranes, while a far smaller number of studies is dedicated to the preparation of RO membranes. Likewise, due to the vast development of current PA TFC RO membranes, performance trade-offs of novel biobased membranes could be expected. Thus, there is still a long way for research in this area to reach the implementation of high-performance biobased RO membrane manufacturing on a large scale.
- Extending the life service time of products is a fundamental concept of the circular economy. Enhancing fouling and chlorine resistance have been identified as the main conditions to extend the service time of current PA TFC RO membranes. Despite the large number of scientific papers focused on the synthesis and modification of PA TFC RO membranes to impair greater fouling and chlorine resistance, a lack of large-scale implementation examples along with long-term stability studies has been identified, hence manifesting a gap between the academic research directions and industrial practical needs. Future works should be devoted to increasing the technology readiness level of long-lasting membrane manufacturing and modification strategies.
- Apart from the membranes, RO module materials design (58% of module weight) should also approach sustainability criteria. Therefore, the replacement or modification of current petroleum-based polymers will be required to improve the biodegradability, reusability, and recyclability of the modules and module materials. However, scarce references in this area can be found in the literature, and thus this research line should be further explored. It has been identified that the current fibreglass casing of the modules increases the labour costs associated with module disassembling, which could limit the implementation of indirect recycling strategies. Furthermore, to date, there are few recycling alternatives for fibreglass materials, mostly related to their use as filler additives. In addition, its high inorganic content (silica glass) with respect to the carbon ratio, means that the material is less suitable as feedstock in waste-to-fuel technologies. Thus, the modification or replacement of the fibreglass casing should be addressed in future RO module design.
- RO membrane reuse and direct recycling technologies have been demonstrated to be technically, economically, and environmentally feasible, and their technological readiness level has already approached the pilot scale validation. Future efforts in these lines should be dedicated to attempting the industrial implementation of validated technologies.
- Recently developed indirect recycling strategies can markedly broaden the applications of recycled membranes and simultaneously enable the individual recycling of plastic components of the RO module, resulting in potential environmental benefits. Up to now, several indirect recycling strategies have been technically validated at a laboratory scale. However, future research should address the potential scalability of each alternative in terms of economic competitiveness and environmental potential. Meanwhile, technological advances in plastic waste recycling (i.e., chemical recycling) would allow the recovery of monomers and other valuable compounds from plastic materials. Thus, advances in this research line (i.e., plastic waste recycling) would favour the potential implementation of indirect recycling strategies.
- Membrane reuse and recycling alternatives are presently technically feasible, have demonstrated economic competitiveness, and could help greatly to reduce the environmental footprint associated with RO membrane-based separation technologies. These alternatives have a promising future in the water and wastewater treatment market. The main limitations encountered for the implementation of membrane reuse and recycling technologies are related to the actual low cost of landfilling, difficulties to bridge the gap between research and industry stakeholders, and the social rejection of second-generation products. However, according to the objectives of the European Commission, increasingly restrictive legislation on unsustainable waste management practices is expected, and among other criteria, a prospective rise in landfilling taxation could be expected. This situation would mean that reuse, recycling and recovering alternatives are even more economically attractive in the near future, thus facilitating their industrial implementation. The future implementation of membrane reuse and recycling technologies would bring several economic, social, and environmental benefits, such as the implementation of low-cost second-generation membranes for the production of high-quality water (e.g., wastewater treatment, desalination).
- Considering the actual situation of the energy sector, waste-to-fuel technologies are increasingly compelling to reduce the volume of waste while producing local energy. Nevertheless, the emissions and residues produced by those alternatives should be carefully evaluated.
- The LCA can estimate the potential sustainability of a technology, identify hot spots, and help in the decision-making process. The results on EoL RO management prioritisation are in good agreement with the waste hierarchy. In this sense, cascade open loop reuse, recycling, and recovering processes are recommended to enable several lifespans of RO elements. In addition, the exploitation of machine learning and artificial intelligence algorithms could revolutionise several sectors and science disciplines, including EoL membrane management processes, allowing for an inexpensive and rapid decision-making process to disclose the most adequate EoL membrane valorisation route, among others.

Overall, the implementation of the eco-design perspective and alternative EoL membrane management options would contribute to the transition toward a greener, competent, and resilient circular economy as a commitment to the objectives of the European Commission set through the Green Deal, the circular economy action plan, and the directive 2008/98/EC on waste. 

## Figures and Tables

**Table 1:** Main characteristics of RO, NF, and UF membranes. Adapted from [4,118].

	Reverse Osmosis (RO)	Nanofiltration (NF)	Ultrafiltration (UF)
Pore size (µm)	<0.001	0.01–0.001	0.1–0.01
Hydraulic permeability (L m^−2^ h^−1^ bar^−1^)	0.05–1.5	1.5–30	10–1000
Working pressure (bar)	20–50	3–20	0.1–5
Separation mechanism	Solution-diffusion model	Sieving and charge effect	Sieving effect
Rejection capacity	Monovalent salts.	Multivalent salts, small organic compounds	Macromolecules, bacteria, viruses

**Table 2:** Possible routes for the valorisation of plastic components from the EoL RO module.

Type of Processing	EoL Component	Processing Method	Recycled Product	Ref.
Direct valorisation	Feed and permeate spacers	Cleaned with water and disinfected	Directly used as geotextile	[116,136]
			Directly used as a mechanical support in membrane preparation.	[137]
	Feed spacer	Cleaned with water and disinfected	Directly used as a mechanical support in membrane preparation.	[137]
			Directly used as turbulence promoters in an ED stack.	[131,132]
Mechanical recycling	Thermoplastics (PP, PET, ABS)	Sorting, shredding, melting, and extruding into new products.	Recycled PP, PET, and ABS, to be extruded into new products.	[108]
			Recycled PP to extrude rigid plastic components of an ED stack	[131]
	Thermosets composites (fibreglass and rubber)	Downsizing by shredding, and granulation to obtain a powder.	Fibreglass powder to be used as a reinforcing additive in concrete, plastics, or roofing products, among others.	[138]
			Rubber powder, to be used as a filler in virgin rubber, concrete, or blended with polymers as a reinforcing material.	[139,140]
Chemical recycling	PP	Ionisation by inductive coupled plasma.	A gas containing 94% of propylene.	[141]
		Oxidative thermolysis.	Acetic acid and other by-products (methanol, formic acid, and propionic acid)	[142]
	PET	Glycolysis Aminolysis	Rigid polyurethanes and polyisocyanurate foams, unsaturated polyester, and epoxy resins. Poly (ester amide)s, polyurethanes, composites, and other materials.	[143]
	ABS	Hydrothermal liquefaction in an alkaline environment.	An oil product composed of oligomers requiring further upgrading.	[144]
	Fibreglass	Chemolysis using subcritical water as a solvent.	Glass fibres and resin monomers	[145]
	Rubber	Devulcanisation by chemical, evulcarmo-mechanical, microwave, or ultrasound processes.	Virgin raw material to be revulcanised into rubber	[139,140]
	RO module	H_2_O_2_-assisted hydrothermal method (pyrolysis)	Oil and gas for fuel and chemical feedstock. Char as carbon precursor for fabricating functional carbon dots.	[146]
